# Adipose-Derived Stem Cells Reduce Lipopolysaccharide-Induced Myelin Degradation and Neuroinflammatory Responses of Glial Cells in Mice

**DOI:** 10.3390/jpm10030066

**Published:** 2020-07-22

**Authors:** Kateryna Yatsenko, Iryna Lushnikova, Alina Ustymenko, Maryna Patseva, Iryna Govbakh, Vitalii Kyryk, Oleg Tsupykov

**Affiliations:** 1 Department of Cytology, Bogomoletz Institute of Physiology NAS of Ukraine, Bogomoletz str., 4, 01024 Kyiv, Ukraine; yatsenkokater@gmail.com (K.Y.); ivlook@ukr.net (I.L.); pma@biph.kiev.ua (M.P.); 2 Cell and Tissue Technologies Department, State Institute of Genetic and Regenerative Medicine NAMS of Ukraine, Vyshgorodska str, 67, 04114 Kyiv, Ukraine; alina.n.ustymenko@gmail.com (A.U.); biomedpost@gmail.com (V.K.); 3 Laboratory of Pathophysiology and Immunology, Chebotarev State Institute of Gerontology NAMS of Ukraine, Vyshgorodska str, 67, 04114 Kyiv, Ukraine; 4 Department of General Practice—Family Medicine, Kharkiv Medical academy of Postgraduate Education, Korchagintsev str., 58, 61000 Kharkiv, Ukraine; irynagovbakh@gmail.com

**Keywords:** models of inflammation, lipopolysaccharide, adipose-derived stem cells

## Abstract

Brain inflammation is a key event triggering the pathological process associated with many neurodegenerative diseases. Current personalized medicine and translational research in neurodegenerative diseases focus on adipose-derived stem cells (ASCs), because they are patient-specific, thereby reducing the risk of immune rejection. ASCs have been shown to exert a therapeutic effect following transplantation in animal models of neuroinflammation. However, the mechanisms by which transplanted ASCs promote cell survival and/or functional recovery are not fully understood. We investigated the effects of ASCs in in vivo and in vitro lipopolysaccharide (LPS)-induced neuroinflammatory models. Brain damage was evaluated immunohistochemically using specific antibody markers of microglia, astroglia and oligodendrocytes. ASCs were used for intracerebral transplantation, as well as for non-contact co-culture with brain slices. In both in vivo and in vitro models, we found that LPS caused micro- and astroglial activation and oligodendrocyte degradation, whereas the presence of ASCs significantly reduced the damaging effects. It should be noted that the observed ASCs protection in a non-contact co-culture suggested that this effect was due to humoral factors via ASC-released biomodulatory molecules. However, further clinical studies are required to establish the therapeutic mechanisms of ASCs, and optimize their use as a part of a personalized medicine strategy.

## 1. Introduction

Neuroinflammation is regarded as one of the factors in the pathogenesis and progression of various neurodegenerative age-associated disorders, such as Parkinson’s disease, Alzheimer´s disease, dementia and multiple sclerosis [1]. It has been shown that brain tissue from patients with neurodegenerative disorders is characterized by pronounced astrogliosis, the activation of microglia and high levels of pro-inflammatory cytokines [2]. Additionally, neuronal cell death may induce neuroinflammation, which by itself may lead to cell death [3]. Lipopolysaccharide (LPS) is often used in different protocols for modeling neuroinflammation associated with neurodegeneration [4]. LPS administration induces in animals’ cognitive impairment, such as decreased locomotion, weight loss, anorexia and others, which are quite similar to clinically relevant symptoms of neurodegenerative disease in humans [5].

Stem cell application is considered as an efficient treatment for neurodegenerative diseases. Cellular regenerative therapy can provide a personalized approach. Individualized stem cell therapy should be adjusted according to the patient specific profile to achieve the best therapeutic results and outcomes. There are several types of stem cells: embryonic, induced pluripotent stem cells and postnatal adult stem cells. Adipose-derived stem cells (ASCs) are adult stem cells and can be regarded as a promising source for personalized cell therapies. ASCs are a population of mesenchymal stem cells (MSCs) obtained from adipose tissues and therefore possess a lot of similar properties to other mesenchymal stem cells [6]. Due to their self-renewal and multilineage properties, ASCs are relatively easy to expand rapidly in culture, and differentiate into several cellular phenotypes in vitro and in vivo.

It has been shown that the transplantation of stem cells derived from adipose tissue attenuates neurological deficits and reduced brain atrophy on the model of hemorrhage insult in rats [7]. ASCs transplantation increases cell proliferation and the number of small vessels, and significantly decreases the area of ischemic brain injury. Human ASCs reduce the destructive changes of neurons in the rat caudate nucleus caused by the injection of 3-nitropropionic acid and decrease edema [8]. The mechanisms of ASCs effect are not fully understood.

Many researchers suppose that the neuroprotective properties of ASCs are realized not directly by the differentiation, but by paracrine signaling through trophic factors and the activation of the recipient cells. Thus, conditioned medium from ASCs culture protects cerebellar granular neurons from apoptosis induced by the activation of caspase-3 [9]. In the model of autoimmune encephalomyelitis, the migration of ASCs in demyelinated areas is accompanied by the increasing of the number of endogenous oligodendrocytes [10].

MSCs have been reported to produce the high level of cytokines such as IGF-1 (insulin-like growth factor-1), VEGF-a (vascular endothelial growth factor-a), SDF-1 (stromal cell-derived factor-1) and erythropoietin, which are involved in the processes of cell proliferation and tissue regeneration [11]. 

Recently, many studies have demonstrated that ASCs display a remarkable ability to modulate inflammatory and immune responses [12,13]. However, it remains unclear if this positive effect is mediated by actual presence ASCs in the tissue, or by distant modulation through the release of the variety of bioactive molecules.

In our study, we used in vivo and in vitro lipopolysaccharide (LPS)-induced neuroinflammatory models to understand the mechanism of the effects of adipose-derived stem cells on neuroinflammation.

## 2. Materials and Methods

### 2.1. Experimental Animals

Adult FVB-Cg-Tg(GFPU)5Nagy/J male mice, transgenic for the GFP gene, were used to obtain adipose-derived stem cells. Mice were maintained under controlled light and environment (12:12 h light/dark cycle, 24 ± 1 °C), and provided with water and food pellets ad libitum. All efforts were made to minimize the number of animals and their suffering during experiments. All procedures complied with the ARRIVE guidelines, were carried out in accordance with the European Convention for the protection of vertebrate animals used for experimental and other scientific purposes (European convention, Strasburg, 1986) and were approved by the Committee for Biomedical Ethics of Bogomoletz Institute of Physiology.

### 2.2. Experimental Groups

Six-day-old (P6) FVB male mice were randomized to one of three groups (8 animals in each group). Group I (control): animals received a single injection of saline. Group II (LPS-treated animals): animals received a single injection of lipopolysaccharide (LPS) and were stereotactically injected with vehicle (DMEM culture medium). Group III (LPS-treated animals and ASCs): animals received a single injection of LPS and were stereotactically injected with ASCs.

### 2.3. In Vivo Model of Neuroinflammation

The peripheral administration of lipopolysaccharide was used to induce neuroinflammation in six-day-old FVB mice (P6). The animals were injected with 0.015 mL endotoxin lipopolysaccharide (LPS, 1 mg/kg) intraperitoneally.

### 2.4. Adipose-Derived Stem Cells Isolation and Culture

Adipose tissue of FVB-Cg-Tg(GFPU)5Nagy/J mice, transgenic for the GFP gene, was used to obtain adipose-derived stem cells. Mice were euthanized by cervical dislocation under avertin anesthesia (300 mg/kg). Subcutaneous inguinal fat pads were harvested under sterile conditions. Isolated adipose tissue was cut into 1–2 mm^3^ pieces, washed with Ca-Mg-free phosphate-buffered saline (PBS) (HyClone, Logan, UT, USA) and digested for 2.5 h at 37 °C with 0.1% collagenase I (Sigma, St. Louis, MO, USA). At the end of this procedure, collagenase was inactivated by adding nutrient medium (DMEM-HG, Sigma) supplemented with 10% of fetal bovine serum (FBS) (HyClone, Logan, UT, USA). After centrifugation, the whole cell suspension for 5 min at 300× *g*, the supernatant was discarded and the pellet (known as stromal vascular fraction-SVF) was resuspended in DMEM-HG medium supplemented with 10% FBS, penicillin 100 U/mL, streptomycin 100 μg/mL, 1:100 non-essential amino acids (all—Sigma-Aldrich, St. Louis, MO, USA) and seeded to T25 culture flask (Sarstedt, Nümbrecht, Germany) at density 4 × 10^3^ cells/cm^2^ for cultivation. The cell suspension was cultivated in complete nutrient medium DMEM-LG, supplemented with 15% FBS, antibiotics (penicillin 100 U/mL, streptomycin 100 mg/mL, (Sigma-Aldrich, St. Louis, MO, USA)), 1:100 non-essential amino acids (Sigma-Aldrich, St. Louis, MO, USA) in a CO_2_ incubator, under conditions of wet air with 5% CO_2_ at 37 °C. The cells from 2–3 passages were used for transplantation. Passaging was carried out at reaching 80% confluence of a monolayer. On the passage two, multiparameter immunophenotyping using flow cytometry as well as directed differentiation into osteogenic and adipogenic directions were performed, as described earlier [14].

### 2.5. Transplantation of GFP-Positive ASCs

The syngeneic transplantation of GFP-positive ASCs suspension into seven-day-old (P7) animals with LPS induced neuroinflammation was performed stereotactically (coordinates: A: 1.5 mm caudal, L: 2.0 mm lateral to bregma, and V: 2.0 mm ventral to the skull surface) monolateral into the right hemisphere of the brain under intraperitoneal calypsol-xylazine anesthesia 24 h after LPS injection. For transplantation, we selected the optimal volume of the culture medium and the dose of cells in it. The optimal dose was—5 × 10^5^ cells in 2 µL medium. 

### 2.6. Non-Contact Organotypic Brain Slice Co-Culture with ASCs

Brain slice cultures were obtained from FVB mice (P7). After the rapid decapitation of animals, the brains were removed, divided into two parts through the median line and cut into 350 microns thick frontal slices with a tissue chopper (McIlwain, Guilford, GB). Each slice included the corpus callosum (from bregma 1.10 to bregma 0.10). We analyzed the area of brain slice culture containing the cortex, corpus callosum and periventricular region.

Brain slices were cultured on the gas-liquid interface on porous Millicell CM inserts (Millipore, Billerica, MA, USA) placed into 6-well culture plates in a CO_2_ incubator with 5% CO_2_ at 35 °C. The culture medium contained 50% MEM, 25% Hanks balanced salt solution, 25% inactivated horse serum, 10 mM Tris, 2 mM NaHCO_3_, 12.5 mM HEPES, 15 mM glucose, 100 U/mL penicillin, 100 µg/mL streptomycin (all—Sigma-Aldrich, St. Louis, MO, USA) at pH 7.2, and was changed on the second day of incubation, and then twice a week. 

For non-contact co-culture, immediately after inducing neuroinflammation with LPS in vitro, inserts with brain slices were transferred to other 6-well plates with earlier adhered ASCs (4 × 10^4^ cells per well). The porous semitransparent membrane of insert was permeable to soluble factors, but prevented direct cell-to-cell contact.

### 2.7. Modeling of Neuroinflammation in Organotypic Brain Slice Culture

In vitro neuroinflammation model was produced by addition of endotoxin lipopolysaccharide (LPS, 100 ng/mL, L4130, Sigma-Aldrich, St. Louis, MO, USA) into culture medium for 48 h, to mimic the inflammation.

### 2.8. Lactate Dehydrogenase (LDH) Assay

The analysis of LDH relative level in the culture medium was performed by colorimetric method. During the injury of cell membrane, cytosolic enzyme LDH releases in the culture medium. The color intensity is directly proportional to the amount of the LDH in the culture medium and inversely proportional to cell viability in the culture.

To determine the changes in the relative amount of cytosolic LDH in culture medium in response to the neuroinflammation modeling or neuroinflammation and ASCs co-culture, 200 µL of culture medium was collected into 24-well plate at 48 h after impact. Samples were collected in duplicates. CytoTox Non-Radioactive Cytotoxicity Assay kit (Promega, Madison, WI, USA) was used for the colorimetric method performed as follows: the reaction was initiated by adding 200 µL of substrate in each well, and incubated at room temperature in the darkness for 30 min; the reaction was terminated by the addition of 200 µL Stop Solution. The optical density of the samples was measured with a spectrophotometer uniSPEC 2 (LLG, Meckenheim, Germany) at wavelength of 492 nm. We determined the average values of duplicates for each well. As controls, we used: 1—culture medium from the wells without organotypic culture (optical density value of which was subtracted from that of obtained from experimental wells); 2—culture medium from the well with untreated neuroinflammation and ASCs cultures. Changes in the relative LDH amount in culture medium was expressed in arbitrary units that represented units of solution optical density, normalized to the tissue area in the respective well. The values were normalized to the control.

### 2.9. Immunohistochemical Staining

Calypsol anesthetized animals were perfused transcardially with 4% formaldehyde solution in 0.1 M phosphate buffer. Coronal sections of the brain were made using vibratome VT1000A (Leica, Nussloch, Germany). A total of 10 coronal sections per mouse were collected. Brain slice cultures were fixed with 4% solution of formaldehyde in 0.1 M PBS. 

Fixed slices and vibratome brain sections were kept in blocking non-specific binding of proteins solution containing 0.1 M PBS (pH 7.4), 0.5% bovine serum albumin (BSA) and 0.3% Triton X-100 (Sigma-Aldrich, St. Louis, MO, USA). Slices and brain sections were incubated in the primary antibodies solution for 12 h at 4 °C. We used the following primary antibodies: anti-GFP (a marker of transplanted cells) 1:7000 (Novus Biologicals, Centennial, CO, USA), anti-MBP (myelin basic protein) 1:1000 (Sigma-Aldrich, St. Louis, MO, USA), anti-Rip (a marker of oligodendrocytes) 1:200 (Abcam, Cambridge, MA, USA), anti-GFAP (a marker of astrocytes) 1:1500 (Dako Cytomation, Glostrup, Denmark) and anti-Iba-1 (a marker of microglia) 1:1000 (Wako, Osaka, Japan). Relevant secondary antibodies, conjugated with fluorochrome AlexaFluor (Invitrogen, Carlsbad, CA, USA), were used to visualize primary antibodies. Stained slices and brain sections were embedded in a drop of Immu-Mount™ mounting medium (Thermo Fisher Scientific, Waltham, MA, USA). Immunohistochemically stained slices and brain sections were studied with confocal scanning microscope FV1000-BX61WI (Olympus, Tokyo, Japan).

Quantitative image analysis was carried out using the ImageJ software, version1.46c (NIH, Bethesda, MD, USA). The intensity and area of fluorescence marker were measured by automatic calculation of the average value of gray within the measurement threshold. The results were presented as integrated density of fluorescence in arbitrary units, which were equal to the fluorescence intensity multiplied by area of fluorescence (excluding the integrated density of the background fluorescence).

### 2.10. Reaching and Retrieving Test

Corticospinal function of animals was assessed using a reaching and retrieving test once a week over a period of 4 weeks, starting after weaning at P28 days [15]. The animals were placed in a plexiglass chamber with a slit of 1.5 cm × 0.5 cm, in which there was a crumb of food as close as 1 cm, to ensure that the animal grabs the food using its legs, rather than its tongue. For 45 min, animals were given 60 attempts to reach and remove the food. The percentage of successful attempts was calculated as following: Successful reaches, % = (number of successful attempts/total number of attempts) × 100, if the animal made 41–60 attempts in 45 min, or successful reaches, % = (number of successful attempts/40) × 100, if the animal has made less than 40 attempts in 45 min.

### 2.11. Statistical Analysis

Data were presented as means ± s.e.m., and analyzed using Student’s *t*-test or ANOVA test, as appropriate. A paired Student’s *t*-test was used to compare two groups of data (i.e., body weight and height). Comparisons among three groups were performed by one-way ANOVA, followed by post hoc multiple comparisons with Tukey’s HSD test using the Origin Pro 8.5 software (Origin Lab. Corp., Northampton, MA, USA). Statistical significance was accepted for *p* values  <  0.05.

## 3. Results

### 3.1. Functional Testing

At the first step of our study, an in vivo model of neuroinflammation was induced by intraperitoneal injection of endotoxin lipopolysaccharide (LPS). LPS-treated animals had a lower weight and height and delayed righting reflex. This reflex corrects the orientation of the body when it is taken out of its normal upright position. 

We investigated the effect of LPS injection and ASCs transplantation on the corticospinal function of experimental animals. Adipose tissue of mice, transgenic by the GFP gene, was used to obtain ASCs. In our previous studies, we performed phenotyping the adipose-derived stem cells culture using flow cytometry. There was detected the high level of CD44, CD73, CD90 expression, while the percentage of cells with hematopoietic markers CD45 and CD117 was less than 2%. The expression of CD34 at the early passages ranged from 8–12%, which is typical for ASCs [14]. According to these results, as well as confirmed potential of directed differentiation into the osteogenic and adipogenic direction, the obtained ASC culture met the minimal criteria for defining multipotent MSCs proposed by the International Society for Cellular Therapy [15]. 

GFP-positive ASCs, second or third passage, were stereotaxic monolaterally injected by a Hamilton syringe into the hemisphere of P7 animals (Group III). Optimal conditions for transplantation, such as volume and the number of cells, were experimentally selected. The optimal dose was two microliters of suspension of GFP-positive ASCs at a concentration of 5 × 10^5^/μL in medium. We performed immunohistochemical analysis with an anti-GFP antibody for tracking the cell fate of transplanted GFP-positive ASCs. It was revealed that, on the 30th day after the transplantation, GFP-positive ASCs were localized in the vicinity of the injection site and did not migrate from the injection zone.

Corticospinal function was estimated by the reaching and retrieving test. The animals were tested once a week over the course of 4 weeks, starting after weaning at P28 [16].

**Figure 1:**
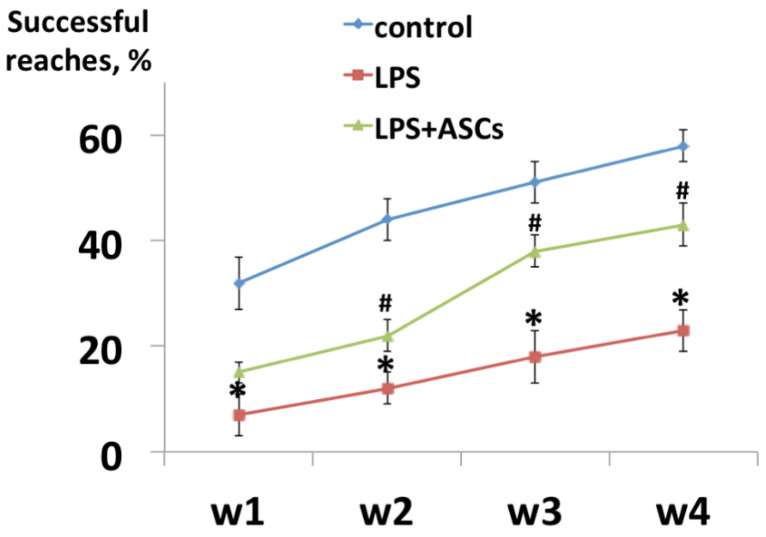
The progression in proficiency in reaching behavior with time in weeks (W). LPS—lipopolysaccharide injected animals (N = 8), ASCs—adipose-derived stem cells treatment (N = 8). *—statistically significant difference compared with control (N = 8) (*p* < 0.05). #—statistically significant difference compared with LPS-injected animals (*p* < 0.05). N = the number of animals in each experimental group.

During the first week of testing, control mice were quick to try reaching for the food, while LPS-treated animals (Group II) were slow to start reaching and had difficulties in grasping the food. As shown in Figure 1, success % in control animals was 32 ± 5%, and in LPS-injected animals—7 ± 2%.

By the second week, control and LPS-treated mice showed increasing levels of proficiency, however, LPS-treated animals were significantly less proficient (*p* < 0.05) at the task than the control (44 ± 4 and 12 ± 3, respectively).

By the fourth week, almost all animals showed improved levels of proficiency that did not significantly change over the three weeks. Success % in control animals was 58 ± 3, and in LPS-treated animals—23 ± 4 (Figure 1).

In the group of animals with ASCs transplantation after LPS injection the recovery of corticospinal function was observed at all time points and by the fourth week the number of successful attempts was 43 ± 4% (Figure 1).

### 3.2. Assessment of Neuroinflammation by Measuring GFAP- and Iba-1-Immunoreactivity

**Figure 2:**
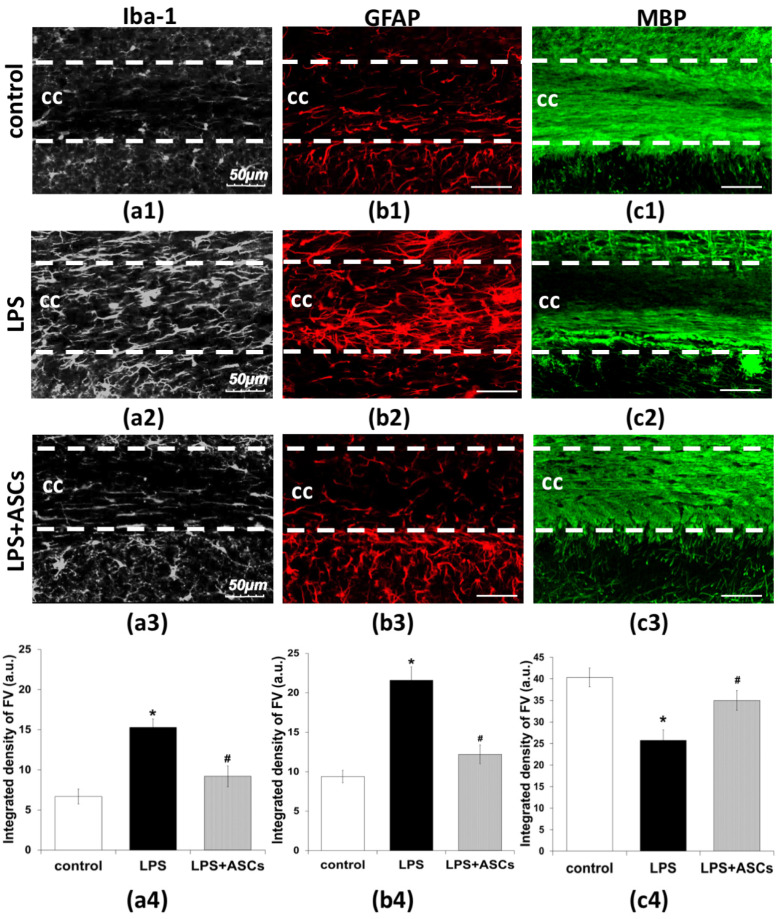
Confocal micrographs of coronal sections of the mice brain stained for Iba-1 (white), GFAP (red) and MBP (green) at day 30 after LPS injection: (**a1**,**b1**,**c1**) control animals (n = 80); (**a2**,**b2**,**c2**) LPS-treated animals (n = 80); (**a3**,**b3**,**c3**) LPS-treated animals and ASCs transplantation (n = 80) (30 day after transplantation). cc—corpus callosum, ASCs—adipose-derived stem cells, LPS—lipopolysaccharide. Scale bar = 50 µm. (**a4**,**b4**,**c4**) histogram of integrated fluorescence density. a.u.—arbitrary units. Note: *—statistically significant difference compared with control (*p* < 0.05), #—statistically significant difference compared with LPS-treated animals (*p* < 0.05). n = the number of analyzed samples.

There are different ways to assess neuroinflammation. We performed immunohistochemical staining with specific markers for microglial activation (Iba-1) and astrocytic response (GFAP). On day 30 after LPS injection, Iba-1 and GFAP immunostaining in the brain slices were found to be significantly increased. More pronounced changes were observed in corpus callosum (Figure 2(a2,b2)). In corpus callosum of control animals, no significant GFAP- and Iba-1-immunoreactivity were detected (Figure 2(a1,b1)).

LPS injection resulted in an increase of the integrated density of corresponding fluorescent signal (Iba-1+, GFAP+), compared with the control group. The integrated fluorescence density of Iba-1-positive microglial cells increased from 6.7 ± 0.9 a.u. in the control group to 15.3 ± 1.1 a.u. in LPS-treated animals (Figure 2(a4)). The integrated density of GFAP-positive signal increased from 9.4 ± 0.8 a.u. in the control group to 21.6 ± 1.7 a.u. in LPS-injected animals (Figure 2(a4)). 

Immunohistochemical analysis revealed that the transplantation of ASCs after LPS injection decreased the Iba-1- and GFAP-immunopositive signaling, in comparison with LPS-treated animals (Figure 2(a4,b4)). The integrated density of Iba-1- and GFAP-positive fluorescence was 9.2 ± 1.3 a.u. and 12.2 ± 1.2 a.u., respectively (Figure 2(a4,b4)).

Next, we performed immunohistochemical staining with specific markers for myelin basic protein (MBP). The degradation of myelin is considered to be an important step leading to neuroinflammation process [17].

The integrated fluorescence density of MBP decreased from 40.3 ± 2.2 a.u. in the control group to 25.8 ± 2.4 a.u. in LPS-treated animals (Figure 2(a4)). The transplantation of ASCs after LPS injection increased the MBP-immunopositive signals compared with LPS-treated animals (Figure 2(c2,c3)) and the integrated density of MBP-positive fluorescence was 35.1 ± 2.3 a.u. (Figure 2(c4)).

Thus, the LPS injection caused myelin basic protein degradation and micro- and astrogliosis, whereas the transplantation of ASCs after in vivo neuroinflammation modeling reduced degradation of MBP and decreased the reactive gliosis.

### 3.3. In Vitro Model of Neuroinflammatory Effects

An in vitro model of neuroinflammation can be used to study the pathogenetic mechanisms of neuroinflammation development, as well as ways of the brain neuroprotection in this pathology.

To test the hypothesis that the neuroprotective properties of ASCs might be also realized by paracrine secretion of various factors, we analyzed the effect of ASCs on nerve tissue 48 h after in vitro modeling of neuroinflammation.

**Figure 3:**
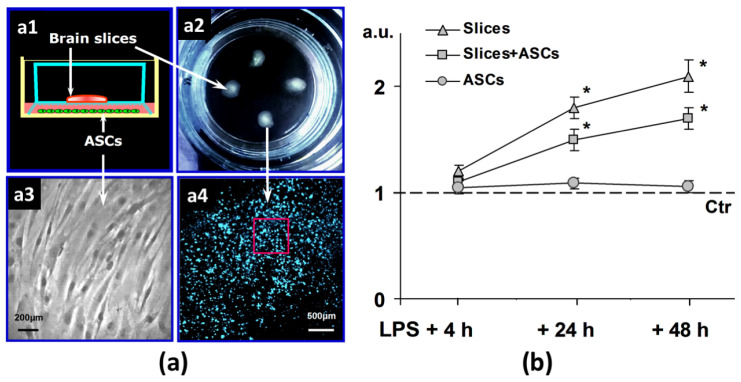
(**a**) The scheme of non-contact organotypic brain slice co-culture with adipose-derived stem cells (ASCs): (**a1**) for non-contact co-culture the inserts with brain slice cultures were placed into the 6-well plates with adherent culture of ASCs; (**a2**) brain slices were transferred onto inserts with porous semitransparent membranes for culturing; (**a3**) the microphotograph of ASC culture, phase contrast, 2nd passage; (**a4**) the analyzed area of slices is shown by the red square. Nuclei were counterstained with Hoechst 33342. (**b**) The relative amount of lactate dehydrogenase (LDH) in the culture medium 4, 24 or 48 h after lipopolysaccharide (LPS) alone or in combination with non-contact ASCs co-culture (n = 8). *—statistically significant difference compared with control (*p* < 0.05). n = the number of slices in the group.

For this purpose, the 2nd passage adhered ASCs (4 × 10^4^ cells per well) were transferred to 6-well culture plates for further non-contact co-cultivation with brain sections cultured on porous semipermeable membranes (Figure 3a).

### 3.4. Viability Assessment by LDH

Spectrophotometric analysis showed that the relative level of LDH in the culture medium was increased by 1.2, 1.8 and 2.1 times, compared with the untreated control after 4, 24 and 48 h, respectively, after the addition of LPS (Figure 3b). 

In contrast, in non-contact co-culture with ASCs, LDH levels in the medium were significantly lower with LPS. It should be noted that ASCs remained viable for 48 h after LPS, since the LDH level did not differ from the control. 

Thus, the data showed that the addition of LPS resulted in significant damage to brain sections, and these effects were reduced in the case of non-contact co-cultivation of slices with ASCs.

### 3.5. Evaluation of Iba-1, GFAP and Rip Positive Glia in Brain Slice Culture

The next step in the study was the estimation of neuroinflammation in organotypic brain slice culture of mice. Immunohistochemical analysis was performed 48 h after treatments, when the impact on the viability (by the LDH levels) was strongly pronounced.

**Figure 4:**
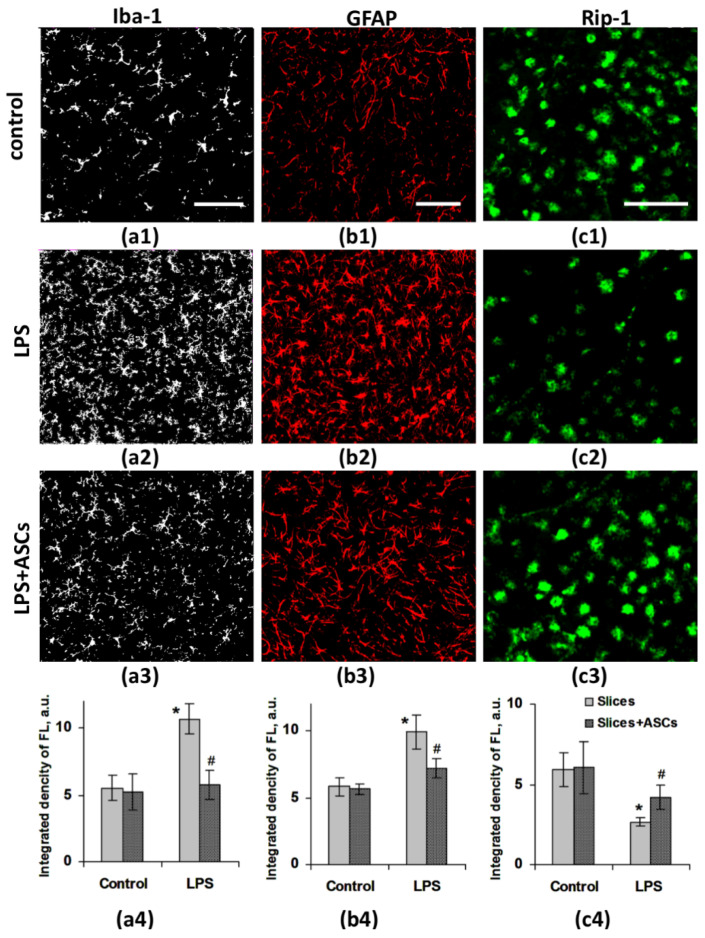
The confocal images of brain slices in control (**a1**,**b1**,**c1**), 48 h after LPS (**a2**,**b2**,**c2**) and LPS + ASCs co-culture (**a3**,**b3**,**c3**). (**a1**–**a4**) microglia (marker Iba-1—white); (**b1**–**b4**) astrocytes (marker GFAP—red); (**c1**–**c4**) oligodendrocytes (marker Rip-1—green) in brain slice culture of mouse. ASCs—adipose-derived stem cells, LPS—lipopolysaccharide. Scale bar—500 μm. (**a4**,**b4**,**c4**) histograms of integrated fluorescence density (n = 8). a.u.—arbitrary units. Note: *—statistically significant difference compared with control (*p* < 0.05), #—statistically significant difference compared with LPS (*p* < 0.05). n = the number of analyzed slices in the group.

The evaluation of Iba-1- and GFAP-positive immunostaining in the brain slice cultures revealed that LPS induced neuroinflammation resulted in an increase of the integrated density of corresponding fluorescent signal (Iba-1+, GFAP+) comparing with control group (Figure 4(a1,a2,b1,b2)).

The integrated fluorescence density of Iba-1-positive microglial cells increased from 5.5 ± 0.9 a.u. in control to 10.7 ± 1.2 a.u. in LPS group. The integrated density of GFAP-positive signal increased from 5.8 ± 0.7 a.u. in control to 9.9 ± 1.3 a.u. for LPS (Figure 4(a4,b4)).

Immunohistochemical analysis revealed that the non-contact co-culturing of brain slices with ASCs after LPS treatment decreased the Iba-1 and GFAP immunopositive signals, compared with LPS treatment only (Figure 4(a3,b3)). The integrated density of Iba-1 and GFAP-positive fluorescence was 5.3 ± 1.3 a.u. and 5.7 ± 0.4 a.u. in control and 5.8 ± 1.1 a.u. and 7.2 ± 0.7 a.u. after LPS, respectively (Figure 4(b4,c4)).

Next, we performed immunohistochemistry by using specific markers for oligodendrocytes (Rip). It was found that the non-contact co-culturing of brain slices with ASCs after LPS treatment increased Rip-immunoreactivity in brain slices compared with LPS group (2.7 ± 0.3 a.u. vs. 4.2 ± 0.7 a.u., respectively), but did not reach control values—5.9 ± 1.1 a.u. (Figure 4(c1–c4)).

Thus, oligodendrocyte damage and reactive astro- and microgliosis were reduced in non-contact co-culture brain slices with ASCs after LPS treatment.

## 4. Discussion

Our data demonstrated that the LPS-induced neuroinflammation resulted in the degradation of myelin basic protein and caused micro- and astrogliosis, whereas the transplantation of ASCs reduced MBP degradation and inhibited the development of neuroinflammation. We also show that the presence of ASCs in the co-culture diminished neuroinflammatory effects improving cell viability, preventing degradation of oligodendrocytes and extensive astro- and microgliosis in the brain slices.

Aiming to achieve the higher fidelity of personalized stem cells therapy, many experiments are carried out to evaluate full potency of adipose-derived stem cells. ASCs not only have a tropism for the injured zone in the brain, but also demonstrate strong immunomodulatory potential suppressing inflammation and enhancing recovery of function [18]. However, it remains unclear if this positive effect is mediated by actual presence ASCs in the tissue, or by distant modulation via releasing bioactive factors. In this study, we used in vivo and in vitro neuroinflammation models to evaluate the protective potential of adipose-derived stem cells.

Adipose-derived stem cells are considered to be a promising source of cells in personalized regenerative medicine, due to their reparative, anti-inflammatory and modulatory properties [19]. ASCs are promising candidates for the treatment for many diseases of the central nervous system, have a strong safety profile and demonstrated good effects in improving functional results through mechanisms implicated in brain plasticity such as neurogenesis, axonal sprouting and angiogenesis [20].

Our study demonstrates that the administration of ASCs to animals with neuroinflammation model contributes to the improvement of behavioral responses, and recovers the cytoarchitectonics of a damaged brain. However, this model does not allow us to distinguish if positive effects are mediated by direct ASCs presence in the affected brain region, and a partial substitution of the damaged cell population or by a distant impact through releasing biomodulators, or both. To answer this question, we used in vitro model of neuroinflammation using organotypic brain slice cultures and investigated protective capacity of ASCs in non-contact co-culture.

Organotypic brain slices culture is a useful object for modeling neuroinflammation and exploring the response of nervous tissue to potential neuroprotective agents including stem cells. The cultured brain slices retain tissue organization, cell-to-cell contacts and a synaptic organization similar to an in vivo environment [21]. The organotypic culture provides an easy access to the extracellular space, allowing better control over experimental conditions and direct impact on tissue by various substances of desired concentration [22]. This experimental system can be useful in researching the contact, as well as the non-contact (humoral), interaction of stem cells with tissue.

For neuroinflammation modeling in vitro on brain slices, we applied endotoxin LPS into culture medium. Cell viability assay revealed that the effect of this harmful factor resulted in the significant cellular damage in 48 h after the LPS treatment (Figure 3). ASCs showed significant protective effect on brain slices at non-contact co-culture increasing the cell survival.

It was shown that glial cells become activated in response to many CNS pathologies, such as trauma, stroke, etc. [23]. The activation of glial cells has been recognized as the primary component and hallmark of neuroinflammation [24]. Astrocytes and microglia are major players in the inflammatory response. It has been demonstrated that toll-like receptor 4 (TLR4), expressed in microglia and astrocytes, plays an important role in neuroinflammation, and can be stimulated by LPS [25]. Being activated, glial cells respond to pro-inflammatory cytokines with an increase in proliferation, change of phenotypes, phagocytosis and release of a battery of pro-inflammatory molecules like iNOS, COX-2, interleukins (IL-1, IL-6) and pro-inflammatory cytokines like TNF-α [26]. The up-regulation of glial intermediate filament is an important step in the glial activation, and the enhancement of these proteins is the best-known hallmark of reactive gliosis [27].

In in vivo neuroinflammation model, we showed that neuroinflammation was accompanied by a pronounced reactive gliosis. Therefore, to assess astro- and microgliosis in in vitro LPS induced neuroinflammation, we performed the immunohistochemical study of the brain slices, using antibodies to the markers of astrocytes (GFAP) and microglial cells (Iba-1). We found that 48h after LPS treatment, astrocytic GFAP and microglial Iba-1 immunoreactivity were significantly increased compared with the control brain slices. The non-contact co-cultivating of brain slices with ASCs following the LPS treatment resulted in the decrease of reactive gliosis.

Earlier studies were based on the hypothesis that the neuroprotective effects of ASCs are caused by the replacement of damaged cells by the differentiation of transplanted ASCs into neurons or glial cells [28]. However, we prefer a model of regeneration induced by the secretion of a variety of cytokines, chemokines, growth and trophic factors [29,30]. At the same time, we do not exclude the possibility of a direct effect of transplanted cells on the recipient’s tissue, which may manifest as cell fusion or differentiation.

There is evidence that the ASCs have neuroprotective potential via modulatory mechanisms by secreting anti-inflammatory molecules [31]. Our data are consistent with previous reports, suggesting that the observed effects are mediated by ASCs-secreted protective factors, as brain slices and ASCs do not have direct contact. Several soluble factors have been reported to involve in ASCs-mediated immunoregulation, such as PGE2, IDO [6] and CX3CL1 [32], which are released by ASCs following stimulation with inflammatory factors.

It was demonstrated that in response proinflammatory cytokines the ASCs secret anti-inflammatory factors such as prostaglandin E2, IL-1ra, TNF-stimulated gene-6 (TSG-6) and IL-10, and modulate a phenotype of microglia toward the anti-inflammatory M2 phenotype and reduce the reactivity of astrocytes [33,34]. Additionally, the MSCs can induce neural progenitor cells differentiation into oligodendrocytes and block differentiation into astrocytes. They facilitate myelination and axon growth by producing miR-146-5p and neurotrophic factors, which contributes to increased expression of nerve growth factor (NGF), brain-derived neurotrophic factor (BDNF), and its high- and low-affinity receptors (TrkA and LNGFR) [34].

In our in vitro model we showed that non-contact organotypic brain slice co-culture with ASCs resulted in the decrease of reactive astro- and microgliosis, and the prevention of degradation of oligodendrocytes and myelin, as in the case of ASC transplantation in vivo. Thus, we suggest that the non-contact paracrine mechanism is central to the neuroprotective and immunomodulatory effects of ASCs.

## 5. Conclusions

Thus, adipose-derived stem cells might be promising therapeutic agents in personalized medicine for the treatment of neuroinflammatory diseases associated with astro- and microglial activation. Further studies are required to explore the precise therapeutic mechanisms of ASCs and optimize their use as a part of a personalized regenerative medicine strategy.
